# Spatial Frequency Shifts From Counterphase Flicker and From Simultaneous Contrast

**DOI:** 10.1177/2041669517707766

**Published:** 2017-05-19

**Authors:** Sae Kaneko, Stuart Anstis

**Affiliations:** Japan Society for the Promotion of Science, Tokyo, Japan; Tohoku University, Miyagi, Japan; University of California, San Diego, CA, USA; University of California, San Diego, CA, USA

**Keywords:** flicker, frequency doubling, simultaneous contrast, spatial frequency

## Abstract

In simultaneous contrast of spatial frequency (SF), a test grating surrounded by a coarser inducing grating looks apparently finer. We combined this effect with another visual illusion; the fact that flickering the inducing grating raises its apparent SF. We found that the inducer’s *apparent*, not physical spatial frequency, drove the simultaneous contrast that it induced into a test grating. Thus, when the inducer was made to flicker, its SF appeared to be higher and consequently, the test’s SF appeared lower than before. This suggests that simultaneous contrast of spatial frequency exists further downstream than the flicker-induced increase in perceived SF.

## Introduction

How we see an object is constantly affected by its spatial context. For example, simultaneous contrast biases a stimulus’s feature towards the complementary of its surround (e.g., a gray object seems reddish on a green background). We call this surrounding stimulus an inducer. Perhaps the best known simultaneous contrast is of brightness/lightness or color (e.g., Heinemann, 1955). However, many other visual features, low level to high level, are susceptible to simultaneous contrast. These include luminance contrast (Chubb, Sperling, & Solomon, 1989; Ejima & Takahashi, 1985), color contrast (Singer & D’zmura, 1994), depth (Graham & Rogers, 1982), orientation (Gibson, 1937; Westheimer, 1990), motion (Anstis & Casco, 2006; Duncker, 1929; Loomis & Nakayama 1973; Tynan & Sekuler, 1975), image blur (Webster, Georgeson, & Webster, 2002), and numerosity/dot density (Durgin, 2001).

Spatial frequency (SF, hereafter) of a grating is one such feature that can show simultaneous contrast. MacKay (1973) first demonstrated in a short paper that a grating appeared finer when surrounded by a coarser grating, and coarser when surrounded by a finer one. Klein, Stromeyer, and Ganz (1974) explored this effect systematically using sinusoidal gratings. They found that the effect was maximal when the orientation of the test and inducing gratings matched and when they differed in SF by 0.5–1 octaves; and the effect decreased when the luminance contrast of the inducer decreased. Successive contrast effect, that is, apparent SF shift due to adaptation, previously reported by Blakemore and Sutton (1969) and Blakemore, Nachmias, and Sutton (1970) shared these properties, which suggests that they share underlying mechanisms (Klein et al., 1974). Lorber and White (1978) replicated the simultaneous effect with brief (10 ms) exposure time of stimuli and additionally demonstrated that if the inducer was presented 75 ms after the test grating the effect disappeared, which suggests fast interaction between the two adjacent areas.

Parker (1981) measured the successive contrast effect of SF. He investigated how the temporal modulation of a grating affected its perceived SF and how that phenomenon in turn affected the successive contrast of SF. Fast (>10 Hz) phase-reversing flickering gratings had been known to increase apparent SF by 30% to 40% (Parker, 1981, 1983; Richards & Felton, 1973; Thompson & Murphy, 1980; Vallam & Metha, 2007; Virsu, Nyman, & Lehtiö, 1974) or sometimes even doubled in SF (frequency doubling; Kelly, 1966; Thompson & Murphy, 1980; Tyler, 1974). Parker asked whether this SF increase affected the successive contrast effect. He found that when an observer adapted to a temporally modulated grating, which appeared higher in SF, the successive contrast effect was dependent on the *apparent* SF of the adaptor; there was no apparent SF change in test stimulus when its SF matched the apparent SF of the adaptor, not the physical SF, and the shift became bigger when the test stimulus SF shifted farther away from the apparent SF of the adaptor.

 On the other hand, Parker found that the change in contrast sensitivity (as opposed to the successive-contrast SF shift) due to adaptation depended on the adaptor’s *physical* SF. Based on this discrepancy between the suprathreshold and subthreshold perception, he supported Klein et al.’s two-stage model of SF processing. As we mentioned, similar properties (orientation selectivity, effect of contrast, etc.) were observed in both simultaneous and successive contrast of SF (Blakemore et al., 1970; Klein et al., 1974). What about the effect of temporal modulation on simultaneous contrast? If the simultaneous and successive contrasts share the same mechanisms, it is likely that we would find the same effect as Parker (1981) in simultaneous version of the effect—namely, if flickering the inducer made it look apparently finer, it would make the surrounded test patch look apparently coarser.

**Figure 1.**
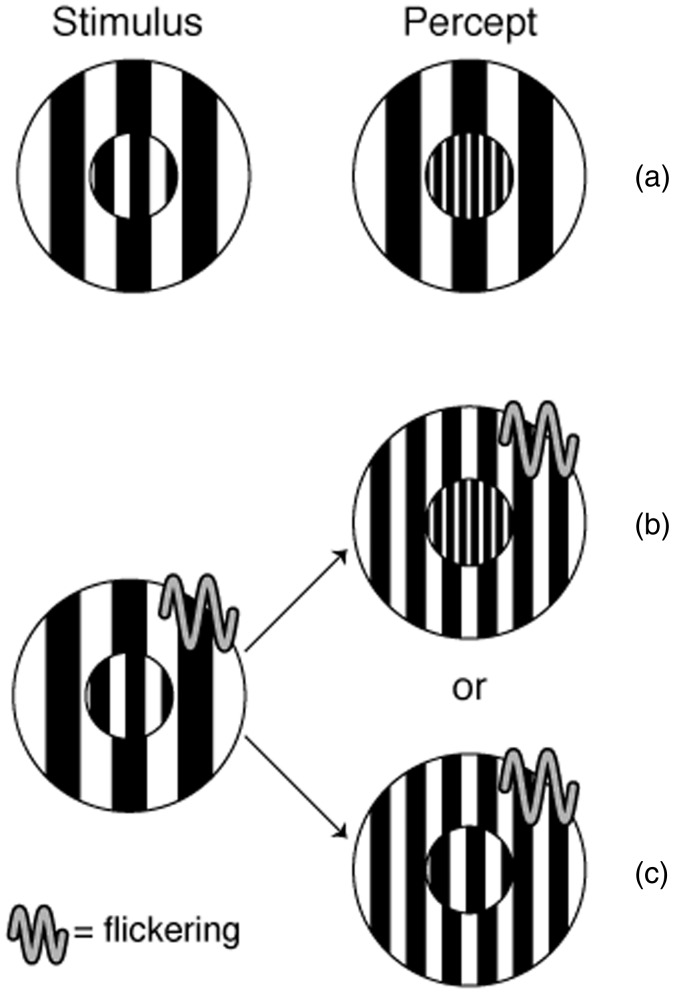
Test appearances predicted when the inducer of same SF is static (a) and flickering (b and c). When the inducer’s SF is lower than that of the test, the test should look finer (a). If the simultaneous contrast depends on the inducer’s physical SF, the test should look the same as in (a) even when the flickering inducer appears finer (b). If the simultaneous contrast depends on the inducer’s apparent SF, the test should, and did, look coarser with the flickering inducer (c) than with the static inducer (a). A wavy line symbol indicates the inducer is flickering.

We examined the simultaneous contrast effect of SF when the inducer continually reversed its spatial phase and therefore appeared higher in SF. In Experiment 1, we first demonstrated that flickering a grating did increase its apparent SF in our particular stimulus setup. In Experiment 2, we examined the simultaneous contrast of SF when the inducer was flickering or static. A test grating surrounded by a coarse inducer looks finer by simultaneous contrast (Figure 1(a)). What happens when the same inducer is flickering (Figure 1(b) and (c))? If the simultaneous contrast depends on the inducer’s physical SF regardless of its change in appearance, then the tests should appear the same whether the inducer is static or flickering (Figure 1(a) and (b)). If the simultaneous contrast depends on the inducer’s apparent SF, then a flickering coarse inducer will look finer and should have much reduced impact on that test’s appearance (Figure 1(c)). As a result, the test should look coarser than with a flickering than with a static inducer (Figure 1(c) and (a)). We confirmed this latter prediction and found that flickering and static inducers of the same SF gave different simultaneous contrast effects.

## Experiment 1

In this experiment, we measured how much the flicker affected the apparent SF of our gratings. Kelly (1966) reported that apparent SF doubling happened for low (<2cpd) SF, fast (>10 Hz) flickering gratings. However, the illusory increase in apparent SF is not restricted to doubling; “partial” (less than double) increase has also been found in similar situations (e.g., Richards & Felton, 1973; Thompson & Murphy, 1980; Vallam & Metha, 2007; Virsu et al., 1974) and the amount of increase depends on the grating’s various parameters. Therefore, it was important to assess how much illusory increase was found in our particular stimulus setup.

### Method

#### Observers

Ten observers participated in this experiment. All had normal or corrected-to-normal visual acuity. All but one of the authors (S. K.) were unaware of the purpose of this experiment.

#### Apparatus and stimuli

All stimuli were presented on a 19-in. CRT monitor (Sony CPD-G400; Sony Corporation, Tokyo, Japan) or on a 22-in. CRT monitor (HP p1230; Hewlett-Packard, California, USA) controlled by a computer (Apple MacPro, Apple Inc., California, USA). Screen spatial resolution was 1280 × 960 pixels (Sony) or 1600 × 1200 pixels (HP). Refresh rate was 100 Hz in both cases. Observers used a chin rest to fix the viewing distance at 52 cm (Sony)/57 cm (HP). Experiments were run in a darkened room.

The stimuli were programmed in MATLAB with Psychtoolbox (Brainard, 1997; Pelli, 1997) routines. The test stimulus was a vertical, luminance-modulated sinusoidal grating seen through a hard-edged circular aperture (radius 4 deg). The contrast of the test grating was set at 100 %, and its SF was either 1.2 or 2 cycles-per-degree (cpd). In half of the trials the test grating’s spatial phase was reversed at 25 Hz (square-wave modulation). In the other half of the trials, the test grating was static. An adjustable, horizontal comparison grating had the same size and luminance contrast as the test stimulus. SF of the comparison grating could be varied from 0.5 to 3 cpd with a 0.025 cpd step. Spatial phases of these gratings were randomized at each trial. The rest of the display was a uniform mean luminance gray of 61 (Sony) or 49 (HP) cd/m^2^.

#### Procedure

An adjustment method was used in this experiment. Observers were asked to adjust the SF of the comparison grating to match that of the test grating while maintaining fixation at the center of the display. The test grating and the comparison were presented side-by-side simultaneously, 5 deg to the left and right of a central fixation point. The comparison grating was always visible while the observer adjusted its SF, and its starting SF was randomized. The test grating was repeatedly presented for 500 ms with 500 ms blank in between. When the observer was satisfied with his adjustment, he hit a designated key to terminate the trial. A 1-s mask of high-contrast random-dots was presented between trials to eliminate any afterimages. Presentation sides of the gratings were counterbalanced across sessions. Each observer made 32 matches per condition.

### Results and Discussion

Our aim was to assess whether flickering a grating increased its apparent SF. Therefore, we took the ratio of SF match of the flickering test grating to that of the static test grating. A value of 1 indicates that flicker did not alter apparent SF. A value greater than 1 indicates that the flickering grating looked finer than the static grating of the same SF.

**Figure 2.**
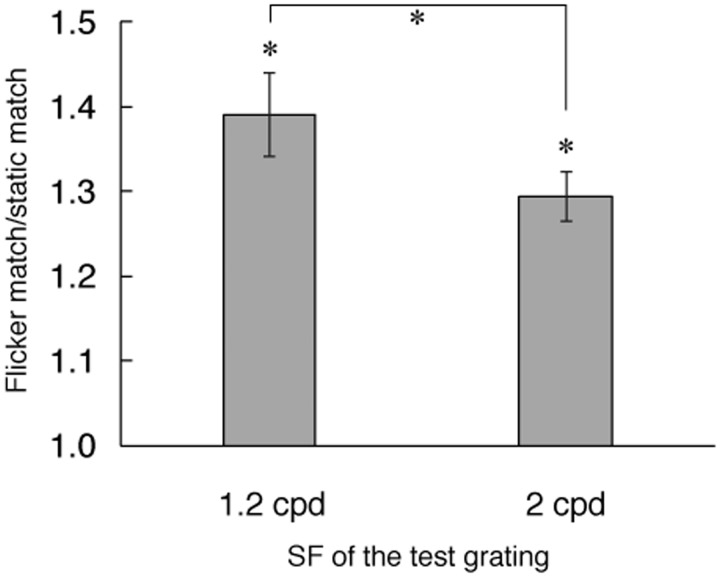
Increase of apparent SF of a flickering grating. A value of 1 means that there was no apparent SF change due to flicker. Mean ± 1 SE for 10 observers. Both increases were significantly greater than 1 (*p* < .05), also increase was greater for 1.2 cpd than for 2 cpd.

Figure 2 shows the average increase ratio for 10 observers. The graph shows that for both SF conditions a flickering grating looked consistently finer than its equivalent static grating, which is consistent with the literature (Parker, 1981, 1983; Richards & Felton, 1973; Thompson & Murphy, 1980; Vallam & Metha, 2007; Virsu et al., 1974). *T* tests show that the flicker/static match ratios are significantly different from 1 (1.2 cpd, *t*_9_ = 7.57, *p* < .05; 2 cpd, *t*_9_ = 9.66, *p* < .05). However, the increase was far less than doubling: Average increase in apparent SF was 39% for 1.2 cpd and 29% for 2 cpd (increase was greater for 1.2 cpd than for 2 cpd, *t*_9_ = 2.89, *p* < .05, which is consistent with the literature; e.g., Parker, 1983). Although for low spatial frequency conditions (<1 cpd) frequency doubling has been reported up to 20 deg eccentricity (McKendrick, Anderson, Johnson, & Fortune, 2003; Vallam, Pataridis, & Metha, 2007), flicker-induced apparent SF increase is greater in central vision for relatively high spatial frequencies (Parker, 1983). Therefore, this smaller effect than doubling in our experiment was not unexpected.

## Experiment 2

Experiment 1 confirmed that fast counterphase flicker raised the apparent frequency of our gratings. In Experiment 2, we examined whether this flicker-induced increase in apparent SF altered the simultaneous contrast effect. Parker (1981) has reported that the successive contrast of SF was based on the *apparent* SF of the adaptors, not their true SF. If his adaptor was coarser than the test grating, but appeared equally fine as the test, then adapting to that adaptor had no effect on the test’s appearance. Our corresponding prediction for simultaneous contrast was described in Figure 1.

**Figure 3.**
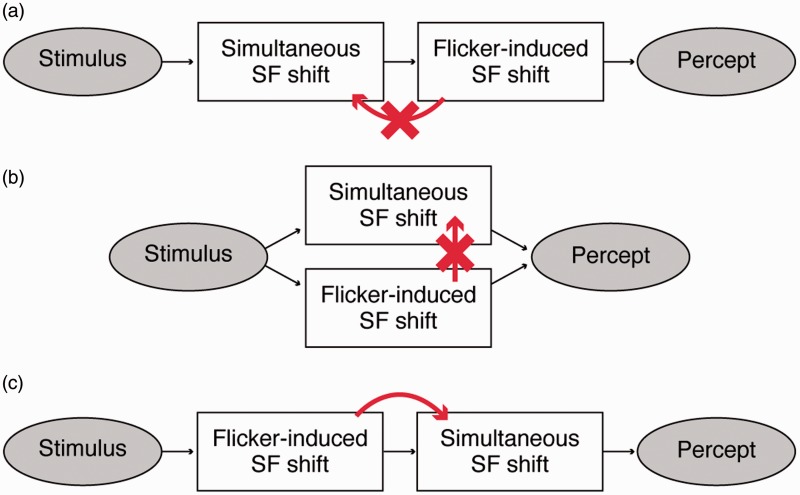
Cartoons showing the possible processing orders of flicker-induced SF shift and simultaneous SF shift. If flicker-induced SF shift follows (a), or is independent from (b), simultaneous SF shift, then whether the inducer is static or flickering should have no effect (crossed-out arrows) on simultaneous SF shift. If flicker-induced SF shift precedes simultaneous SF shift (c), then the change in inducer appearance might affect simultaneous SF shift (red arrow). Our results were consistent with (c).

Our aim was to establish whether the flicker-induced SF shift was processed before or after simultaneous contrast SF shift. If flicker-induced SF shift occurs after (Figure 3(a)) or independently from simultaneous SF shift (Figure 3(b)), any appearance change of the inducer should not affect simultaneous SF shift. Flicker-induced SF shift could affect simultaneous SF shift only if it occurred *before* the simultaneous SF shift (Figure 3(c)).

### Method

#### Observers

Same as Experiment 1.

#### Apparatus and stimuli

The apparatus was identical to Experiment 1. Stimuli were also similar except for the following. A test grating, whose apparent SF the observers judged, was a 2 cpd sinusoidal vertical grating (luminance contrast 100 %) within a circular aperture (now radius 2 deg). Except for the control condition (no surround), the doughnut-shaped grating (inner radius 2 deg, outer radius 4 deg; “inducer” hereafter) was also presented with the test grating. SF of the inducer was either 1.2 (“coarser” condition) or 2 (“same” condition) cpd. The inducer was vertical and was either flickering (phase-reversed) at 25 Hz or static. To ensure that the observers could segregate the test grating and the inducer, when the two gratings had the same SF (i.e., 2 cpd), they were fixed at a relative spatial phase difference of two-third cycle. The comparison grating was a horizontal sinusoidal grating with 4 deg radius circular aperture as in Experiment 1, and its SF was varied from −0.5 to + 0.5 octave around 2 cpd (1.4–2.8 cpd) in 11 steps (equally spaced along a logarithmic scale).

#### Procedure

The method of constant stimuli was used in this experiment. A test grating and a comparison grating were presented side-by-side simultaneously for 1,000 ms, 5 deg to the left and right of a central fixation point. After the gratings disappeared, a binary random-dot noise field with a fixation point was presented while waiting for an observer’s response. Observers were asked to judge which of the two gratings, the test grating or the comparison grating, was finer (i.e., higher in SF) while ignoring the inducer. Response was made by hitting one of two designated keys on the keyboard. There were 55 conditions in total (5 inducer conditions including the control condition ×11 comparison SF conditions). All conditions were run in one session in a random order. Presentation sides of the gratings were counterbalanced across sessions. Each observer made 24 matches per condition.

### Results and Discussion

**Figure 4.**
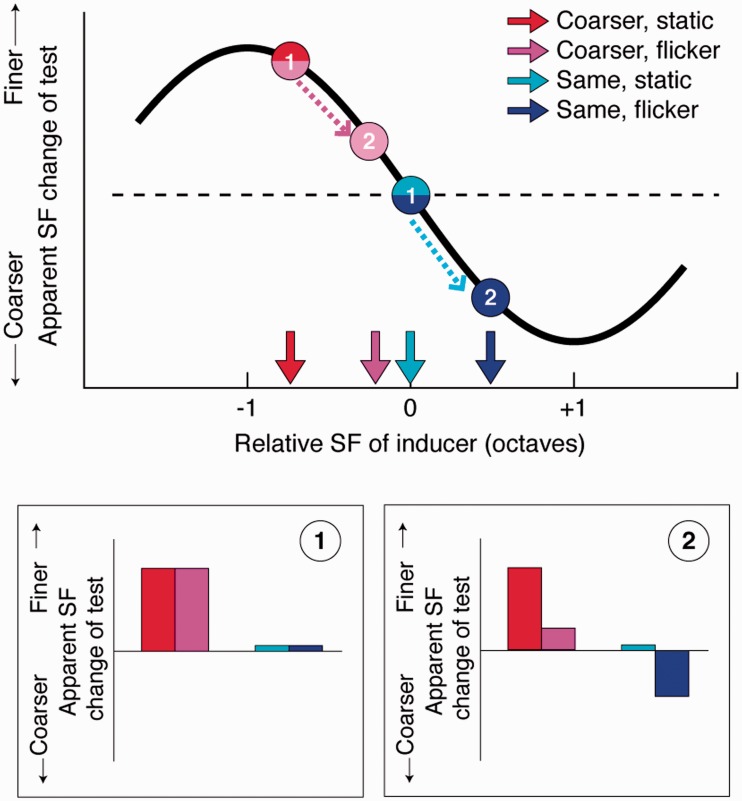
Predicted simultaneous SF shift size for different conditions. Top, Arrows along the horizontal axis show the apparent SF of the inducers for four conditions (assuming the +40% increase of the apparent SF increase due to flicker). Black S-shaped curve is derived from Klein et al. (1974) data, showing the relationship between the simultaneous SF shift and the inducer’s SF. The effect peaks when the inducer’s SF is approximately one octave away from the test SF. Note that the apparent SF shift by flicker was smaller than one octave shift. Bottom: If flicker-induced SF shift follows or is independent from simultaneous SF shift (Figure 3(a) and (b)), the *physical* SF of the inducer should determine the simultaneous SF shift size, therefore there should be no difference between inducers of the same SF (top and bottom, #1). If flicker-induced SF shift does affect and determine the simultaneous SF shift size, then the *apparent* SF of the inducer matters and flickering inducer should make the test coarser than the static equivalent (top and bottom, #2). Therefore, “coarser, flicker” should yield closer-to-zero effect and “same, flicker” should yield non-zero, coarsening effect. The critical point here is that the test looks always *coarser* when surrounded by flickering inducer than with static inducer of the same SF in this prediction.

First, we show our prediction in Figure 4. If flicker-induced SF shift in the inducer does *not* affect simultaneous SF shift, there should be no difference in simultaneous SF shift between the inducers of the same SF, that is, between “coarser, flicker” and “coarser, static”, and between “same, flicker” and “same, static” (Figure 3(a) and (b): Figure 4, bottom 1). If, on the other hand, flicker-induced SF shift in the inducer *does* affect simultaneous SF shift, and this apparent SF of the inducer determines the simultaneous SF shift in both “coarser” and “same” conditions, then the flickering inducer should make the test *coarser* than its equivalent static inducer (Figure 3(c); Figure 4, bottom 2).

The point of subjective equality (PSE) was estimated per condition by fitting a psychometric curve to each observer’s data. Cumulative Gaussian curves were fitted using routines provided by Foster and Bischof (1997). PSE was defined as the SF of a comparison grating when it was judged as finer than the test with a 50% probability.

**Figure 5.**
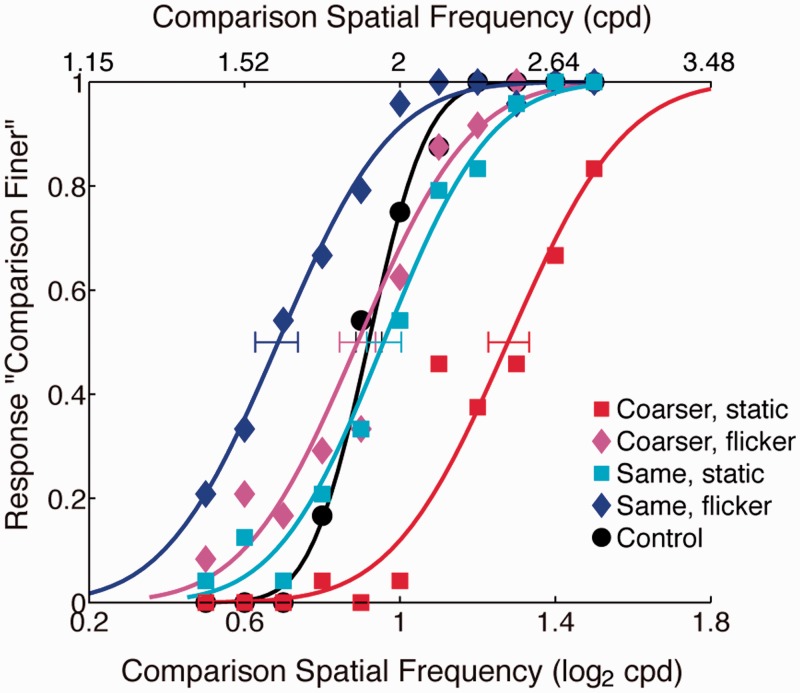
Psychometric curves for five inducer conditions in Experiment 2 (one naïve individual’s data). Data points show rates of an observer’s judging comparison gratings as finer than the test (2 cpd). Bluish colors are for conditions with same frequency (2 cpd) inducers. Reddish colors are for conditions with coarser (1.2 cpd) inducers. Black curve is for the control condition with no inducer. Rightward shift of a curve means the test looked finer. Error bars denote 95% CI calculated from 5,000 bootstrap replications.

Figure 5 shows psychometric curves from a representative naïve observer. PSE of the control condition, where there was no inducer presented with the test grating, falls near veridical 2 cpd point but with a leftward shift indicating a slight bias in this observer. We normalized the data to eliminate such biases by subtracting the control PSE from the experimental PSEs. We defined this difference from the control PSE as the apparent SF shift size.

**Figure 6.**
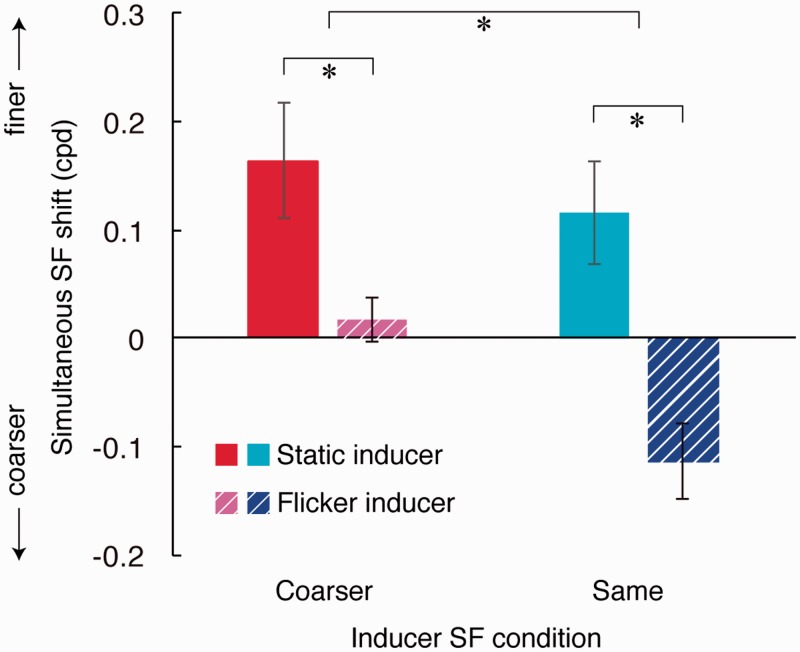
Simultaneous SF shift with various inducers in Experiment 2 (mean ± 1 SE, group data). A positive (negative) value means inducers made the test look finer (coarser). Significant differences are marked with asterisks (*p* < .05). Results show that flickering the inducer (which made it look finer) made the test gratings look coarser.

These apparent SF shifts for four conditions are shown in Figure 6 (mean of 10 observers). Two-way analysis of variance was performed on these apparent spatial frequencies; the result revealed that there was a significant main effect of flicker (*F*(1, 9) = 13.09, *p* < .05) and of inducer SF (*F*(1, 9) = 5.45, *p* < .05), but no significant interaction (*F*(1, 9) = 1.84, *p* > .05). This suggests that the flickering inducer made the test appear lower in SF than the equivalent static inducer. Independent *T* tests showed that the apparent SF shift was significantly different from zero with coarse static inducer (*t*_9_ = 2.93, *p* < .05) and same flicker inducer (*t*_9_ = −3.04, *p* < .05) but not for other two conditions (coarser flicker inducer, *t*_9_ = 0.84, *p* > .05; same static inducer, *t*_9_ = 2.23, *p* > .05). Although nonsignificant, the fairly large shift in apparent SF by the “static, same” inducer was unexpected. We will discuss this effect in our control experiment later.

The positive SF shift with coarser static inducer means that the test appeared finer when surrounded by a coarser grating, which confirms the effect of simultaneous contrast of SF (Klein et al., 1974; Lorber & White, 1978; MacKay, 1973). If this simultaneous SF shift occurs depending on the inducer’s physical SF, we would expect the same amount of positive shift in apparent SF with flickering coarser inducer. However, the flickering coarser inducer did not shift the apparent SF of the test grating. According to Experiment 1, in this condition the inducer should have appeared as ∼1.7 cpd, closer to the test SF than its true SF (1.2 cpd). Different amount of simultaneous SF shift with static and flickering inducers implies that the simultaneous contrast is dependent on the *apparent*, not the physical SF of the inducer. This finding of ours is consistent with the hypothesis that the simultaneous contrast occurs after the flicker-induced SF shift.

We shall now consider, and reject, a possible objection. Our last results could be explained by the effective contrast reduction of the inducer. Contrast sensitivity falls greatly at higher (>10 Hz) temporal frequency (e.g., Robson, 1966), and fast flickering gratings could appear lower in contrast (Georgeson, 1987). Although we did not measure the apparent contrast of the inducer, this might have been the case for our stimuli. The simultaneous spatial frequency contrast effect decreases as the luminance-contrast of the inducer decreases (Klein et al., 1974). Therefore, an alternative hypothesis to explain our results would be that the flickering inducer had lower effective contrast and therefore induced less simultaneous contrast than did the static equivalent.

However, data from “same” inducer conditions clarify this point. “Same” inducers should have little or no simultaneous contrast effect, extrapolating from data by Klein et al. (1974). Indeed, although there was a positive trend in shift, our static same-inducer condition yielded no significant shift in apparent SF (the possible explanation of this shift is discussed in the following section). If the physical spatial frequency drives simultaneous contrast, the flickering “same” inducer should also bring about zero shift. Alternatively, if the effective contrast of the inducer drives simultaneous contrast, then the flickering “same” inducer would give an even smaller shift. Our results show that the flickering “same” inducer in fact significantly reduced the SF of the test, that is, made it look coarser. This implies that the simultaneous contrast depends upon the inducer’s apparent SF, which in this condition should be finer than the test due to flicker (should be ∼2.6 cpd based on Experiment 1, see also Figure 4).

#### Control experiment: The effect of aperture size on apparent SF

In Experiment 2, there was a fairly large shift in apparent SF in the “same, static” condition, which was not expected. This suggests that compared to a large (4 deg radius) horizontal comparison grating, a small (2 deg radius) vertical grating appeared to be finer when it was embedded in a large (4 deg radius) vertical grating of the same SF than when it was surrounded by a uniform gray field. In Klein et al. (1974), the zero crossing of the S-shaped function of the simultaneous SF shift, that is when the test grating was not surrounded by finer grating nor by the coarser grating, was when the test was surrounded by uniform field, not by the grating of the same SF. We extrapolated and predicted that the inducer of the same SF should have no effect on apparent SF of the test, but it seems that there is a difference in “no surround” and “same (SF) surround.”

In this control experiment, we examined the possible effect of the aperture size on apparent SF to account for the mysterious SF shift of “same, static” condition in Experiment 2. Aperture size is known to affect perithreshold and suprathreshold perception in different visual features (one example is the speed perception; Brown, 1931) including spatial pattern perception (Masin, 1980; Vicario, 1972). Therefore, we speculated that it was possible that the small test grating embedded in a larger grating of the same SF (=“same, static” stimulus) was treated as one large grating, and that aperture size difference affected the apparent SF.

**Figure 7.**
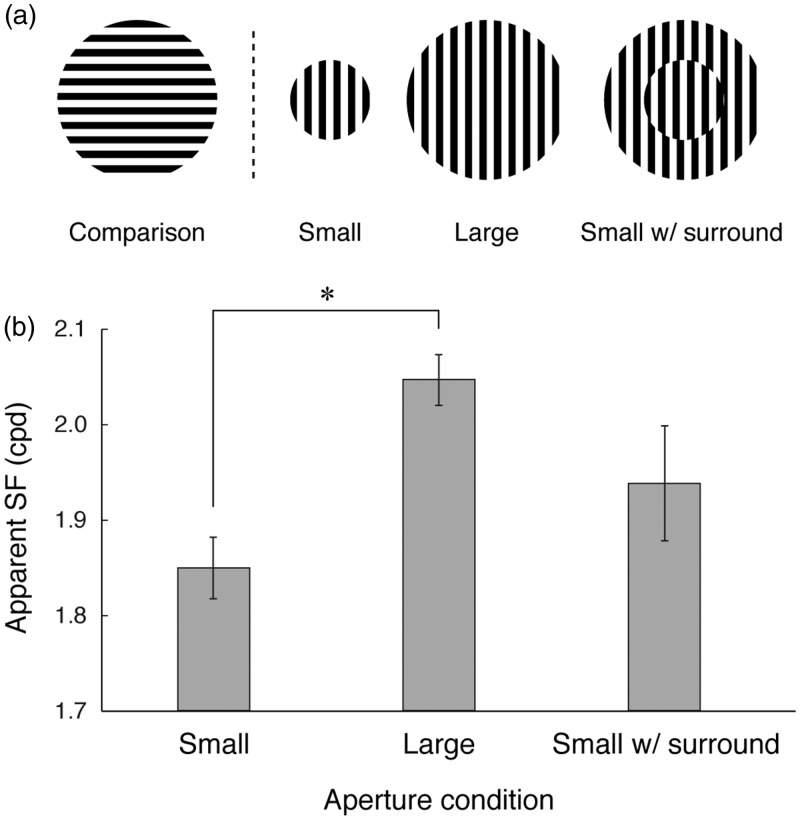
(a) Schematic description of the three aperture conditions along with the comparison stimulus. Circular aperture size was 2 deg radius for “Small” and 4 deg radius for “Large”, the surround for “Small with surround”, and the comparison. For “Small with surround”, the relative spatial phases of the test (small) and the surround was fixed to two-third cycle difference to visually segregate the two areas. (b) Measured apparent SF of the test grating (2 cpd). Mean of five observers with ± 1 SE.

The stimuli and the procedure were the same as Experiment 2 except for following minor modifications. The test grating conditions were now only three, “Small” (grating with 2 deg radius aperture), “Large” (grating with 4 deg radius aperture), and “Small with surround” (grating with 2 deg radius aperture embedded in a 4 deg radius grating) (Figure 7(a)). The grating itself was static and 2 cpd in all conditions. The SF of comparison was varied in nine steps from −0.4 to +0.4 octaves around 2 cpd. Observers were instructed to focus on the center of the test pattern. Five observers (including one of the authors) participated.

Results are shown in Figure 7(b). There was a clear effect of the aperture size on the apparent SF. One-way analysis of variance revealed a significant effect of the aperture size condition (*F*(2, 8) = 4.61, *p* < .05) and the post-hoc comparison showed a difference between “Small” and “Large” (*p* < .05, Bonferroni). Our result indicates that the grating looked finer with a larger aperture, which is consistent with the simultaneous size contrast effect (as in Ebbinghaus illusion; see also Vicario, 1972). Therefore, we confirmed the effect of the aperture size on the apparent SF of the grating contained. “Small with surround”, which was identical to “Same, static” in Experiment 2, fell between “Small” and “Large.” From this, we inferred that despite our instruction to focus on the center of the test grating, the stimulus was sometimes, but not always, treated as one large grating and that resulted in the seemingly inexplicable shift in apparent SF in “Same, static” condition in Experiment 2.

## General Discussion

We examined the simultaneous contrast of SF when the inducer’s physical and perceptual SF differed. We relied on the fact that fast phase-reversal flicker makes a grating appears higher in SF (Kelly, 1966; Richards & Felton, 1973; Thompson & Murphy, 1980; Vallam & Metha, 2007; Virsu et al., 1974). In Experiment 1, we confirmed that our gratings did appear higher in SF, less than doubling (Kelly, 1966) but showing a significant ∼30% increase (Parker, 1983). Experiment 2 showed that when the inducer and test had the same physical SF, flickering the inducer changed the amount of simultaneous contrast. Simultaneous contrast depended on the inducers’ *apparent* spatial frequencies, not their physical spatial frequencies, suggesting that the flicker-induced SF shift occurs *before* the simultaneous SF shift.

Our finding that simultaneous contrast effect depends on apparent SF is very similar to what Parker (1981) found in successive contrast of SF. Klein et al. (1974) suggested that simultaneous and successive contrast of SF may share similar mechanisms, and the similarity between our results and those of Parker (1981) adds evidence to this hypothesis.

Frequency doubling effect has been generally regarded as a result of nonlinear rectification at retinal level processing (Tyler, 1974). However, a physiological study by White, Sun, Swanson, and Lee (2002) failed to find assumed substrates in macaque retinal cells and suggested more central processing of the illusion. A rectification theory of frequency doubling fails to explain the cases of partial increase of apparent SF or the similar partial effect brought by drifting motion (demonstrated by Parker, 1981, 1983). Unless the frequency doubling is an independent phenomenon unrelated to the partial increase (and indeed Parker, 1983, suggested as such), the rectification theory has to be challenged. Virsu et al. (1974) suggested as an alternative theory that the temporal modulation of the stimulus alters the tuning of SF tuned units in the way that the overall group activity of units would signal higher SF. This would explain both the partial increase and doubling. On the other hand, physiological studies have shown that SF tunings of cells in V1 and V2 were basically independent of stimulus temporal frequency (Foster, Gaska, Nagler, & Pollen, 1985).

Studies on the mechanisms of simultaneous contrast of SF shift are surprisingly sparse. Blakemore et al. (1970) suggested lateral inhibition between SF tuned channels to explain their well-known adaptation to SF (also Blakemore & Sutton, 1969). Since both the simultaneous and successive effects are orientation selective (Blakemore et al., 1970; Klein et al., 1974), Blakemore et al. (1970) suggested that the adaptation site was not below the cortex, where orientation selectivity can first be found (e.g., Hubel & Wiesel, 1962). Klein et al. (1974) pointed out the fundamental difference in effect of neighboring gratings on the test’s threshold and suprathreshold perceptions, and they proposed a modification to Blakemore’s theory; a two-stage model. The model basically agreed with the concept of inhibition between SF tuned channels/units but separated them into two hierarchically different stages to compromise with the difference of effect from the surround. There are “analyzers” tuned to various orientations and spatial frequencies that feed into detection mechanism (for detection) and to SF tuned “integrators” (for perception). These integrators have inhibitory connections with neighbors and the simultaneous contrast effect occurs here. Parker (1981, 1983) agreed with this two-stage model based on his result showing different effect of adaptation on sub- and suprathreshold perception. To our knowledge, there is no study that directly shows that inhibitory connection between neurons in any visual area correlate with the suprathreshold perception of SF, as these papers proposed.

In summary, our study showed that static versus flickering inducers of same SF could produce different amounts of simultaneous contrast, depending on the *apparent* SF of the inducers. This should help us figure out the relative orders of the processing stages of the two illusions (Figure 3). The dependency on apparent SF that we found should happen only if the apparent SF change of the inducer occurs *before* the simultaneous contrast. Thus, our results suggest that the flicker-induced SF shift is processed at an earlier neural stage than simultaneous contrast of SF. This conclusion agrees with the results shown by Parker (1981) with successive contrast of SF.
